# Biomarkers in 5q-associated spinal muscular atrophy—a narrative review

**DOI:** 10.1007/s00415-023-11787-y

**Published:** 2023-06-08

**Authors:** H. S. Lapp, M. Freigang, T. Hagenacker, M. Weiler, C. D. Wurster, René Günther

**Affiliations:** 1 Department of Neurology, University Hospital Carl Gustav Carus at TU Dresden, Fetscherstraße 74, 01307 Dresden, Germany; 2 Department of Neurology and Center for Translational Neuro- and Behavioral Science (C-TNBS), University Medicine Essen, Essen, Germany; 3 Department of Neurology, Heidelberg University Hospital, Heidelberg, Germany; 4 Department of Neurology, University Hospital Ulm, Ulm, Germany; 5 German Center for Neurodegenerative Diseases (DZNE) Ulm, Ulm, Germany; 6 German Center for Neurodegenerative Diseases (DZNE) Dresden, Dresden, Germany

**Keywords:** Spinal muscular atrophy, Biomarker, Disease management, Muscle integrity

## Abstract

5q-associated spinal muscular atrophy (SMA) is a rare genetic disease caused by mutations in the *SMN1* gene, resulting in a loss of functional SMN protein and consecutive degeneration of motor neurons in the ventral horn. The disease is clinically characterized by proximal paralysis and secondary skeletal muscle atrophy. New disease-modifying drugs driving *SMN* gene expression have been developed in the past decade and have revolutionized SMA treatment. The rise of treatment options led to a concomitant need of biomarkers for therapeutic guidance and an improved disease monitoring. Intensive efforts have been undertaken to develop suitable markers, and numerous candidate biomarkers for diagnostic, prognostic, and predictive values have been identified. The most promising markers include appliance-based measures such as electrophysiological and imaging-based indices as well as molecular markers including SMN-related proteins and markers of neurodegeneration and skeletal muscle integrity. However, none of the proposed biomarkers have been validated for the clinical routine yet. In this narrative review, we discuss the most promising candidate biomarkers for SMA and expand the discussion by addressing the largely unfolded potential of muscle integrity markers, especially in the context of upcoming muscle-targeting therapies. While the discussed candidate biomarkers hold potential as either diagnostic (e.g., SMN-related biomarkers), prognostic (e.g., markers of neurodegeneration, imaging-based markers), predictive (e.g., electrophysiological markers) or response markers (e.g., muscle integrity markers), no single measure seems to be suitable to cover all biomarker categories. Hence, a combination of different biomarkers and clinical assessments appears to be the most expedient solution at the time.

## Introduction

5q-associated spinal muscular atrophy (SMA) is a rare genetic disease caused by homozygous deletions or compound heterogeneous mutations in the *SMN1* gene, resulting in a loss of functional survival motor neuron (SMN) protein [1]. The SMN protein is essential for spinal alpha motor neuron vitality, and its depletion leads to degeneration of these motor neurons with secondary skeletal muscle atrophy, which clinically manifest in proximal paralysis. In much lesser quantities, full-length SMN production is also provided by the *SMN2* gene, centromeric copies of *SMN1* [2]. However, due to a point mutation at position 6 of exon 7 in *SMN2* and an intronic splicing silencer N1 (ISS-N1), splicing of exon 7 of *SMN2* pre-mRNA is altered leading to production of only low levels of SMN protein [3]. The clinical phenotype of patients with SMA is most likely multifactorial but generally correlates with *SMN2* copy numbers and the amount of the resulting functional SMN protein (SMN-P) [4–6].

While there were no specific treatment options for SMA for years, some promising therapeutic options have risen in the past decade. Currently approved therapeutic strategies aim at increasing the amount of functional SMN protein by either SMN1 gene replacement (onasemnogene abeparvovec, [7–9]) or SMN2 splice modulation (nusinersen [10–14], risdiplam [15, 16, 17–19]) and have shown beneficial therapeutic response [20–22]. SMN-independent approaches are currently tested in ongoing randomized trials. Apitegromab is a myostatin activation inhibitor that has shown to stabilize and improve motor function in patients with SMA additionally to nusinersen in a phase 2 study [23], with an increase in the Hammersmith functional motor scale expanded (HFMSE [24]) of ≥ 1 point in ≥ 40% of the patients and ≥ 3 points in ≥ 20% of the patients. In mouse models, BIO101, a Mas receptor activator, has shown beneficial effects on fatigue levels and motor function [25]. Reldesemtiv is a fast skeletal muscle activator that selectively binds to the fast skeletal troponin complex and sensitizes it to calcium [26], thereby increasing muscle strength relative to the neuronal input. In a phase 2 study, it has proven to increase the slow vital capacity and the six-minute walk test (6MWT) in SMA patients [27]. Amifampridine is a voltage-dependent potassium channel blocker that has proven to empower neuromuscular transmission and muscle function in Lambert–Eaton myasthenic syndrome [28] and most recently in ambulatory SMA type 3 patients [29].

**Fig. 1:**
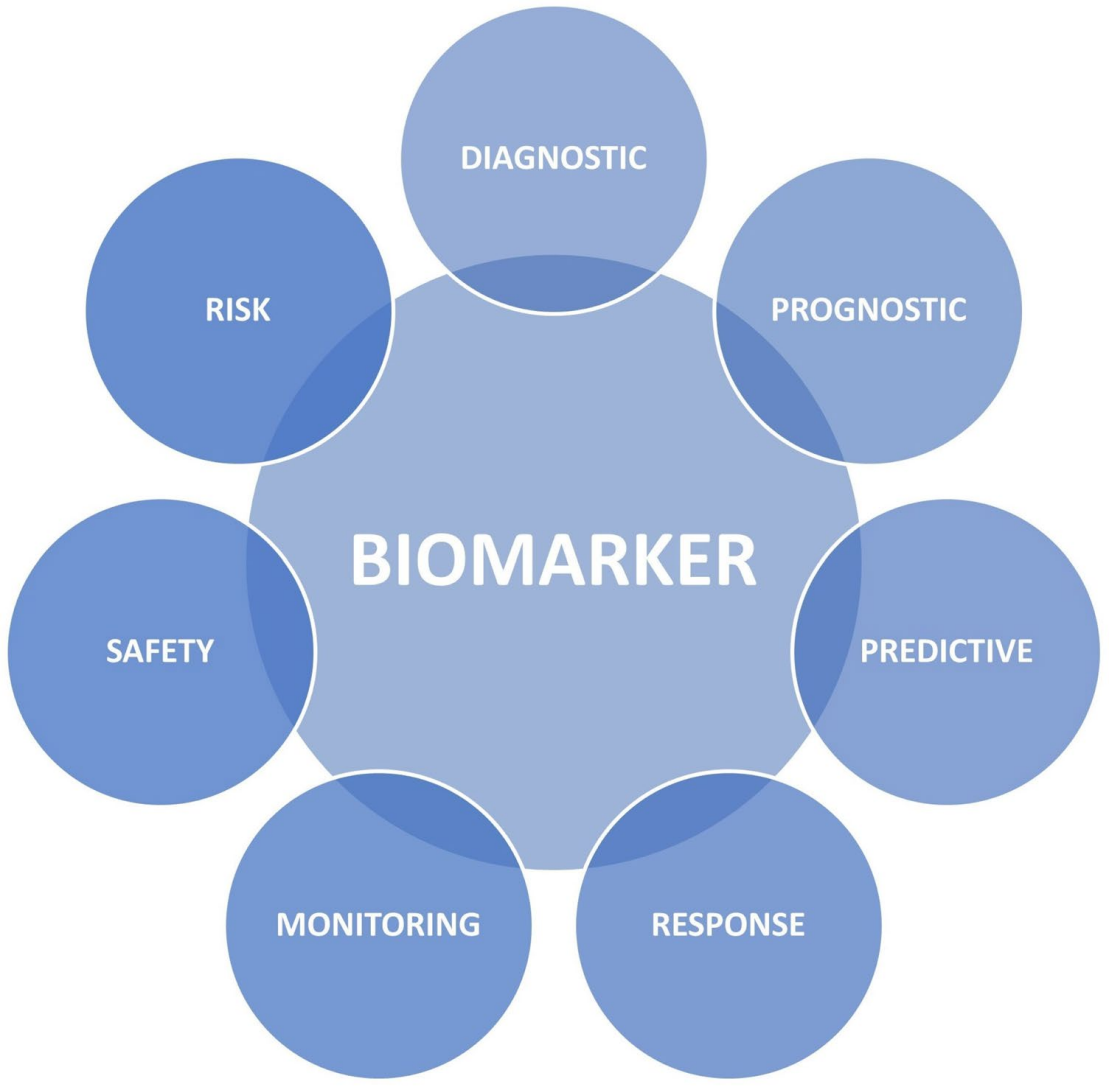
Biomarker categories according to the BEST resource (FDA)

As more therapeutic strategies evolve, there is a rising need for biomarkers, especially to stratify for treatment eligibility and to monitor treatment efficacy. Biomarkers can be classified in different categories, depending on their purpose (see Fig. 1). The U.S. Food and Drug Administration (FDA) has formed a working group that has defined seven main categories of biomarkers [30]. These main categories are (1) diagnostic biomarkers that help to detect or confirm the presence of a disease or to identify individuals with a subtype of the disease, (2) prognostic biomarkers that identify the likelihood of a clinical event or disease progression in an individual with the disease, (3) predictive biomarkers that identify individuals who are more likely to experience an effect from a certain medical product, (4) response biomarkers that show that a biological response has occurred in an individual exposed to medical treatment (e.g., pharmacodynamic biomarkers that measure the biological activity of the medical product, not necessarily drawing conclusions to clinical outcome) (5) monitoring biomarkers for repeated assessment of the status of a disease or the effect of medical treatment, (6) safety biomarkers that indicate toxicity of medical treatment, and (7) risk biomarkers that reflect the potential for developing a disease in an individual who does not currently have the disease [30, 31].

**Fig. 2:**
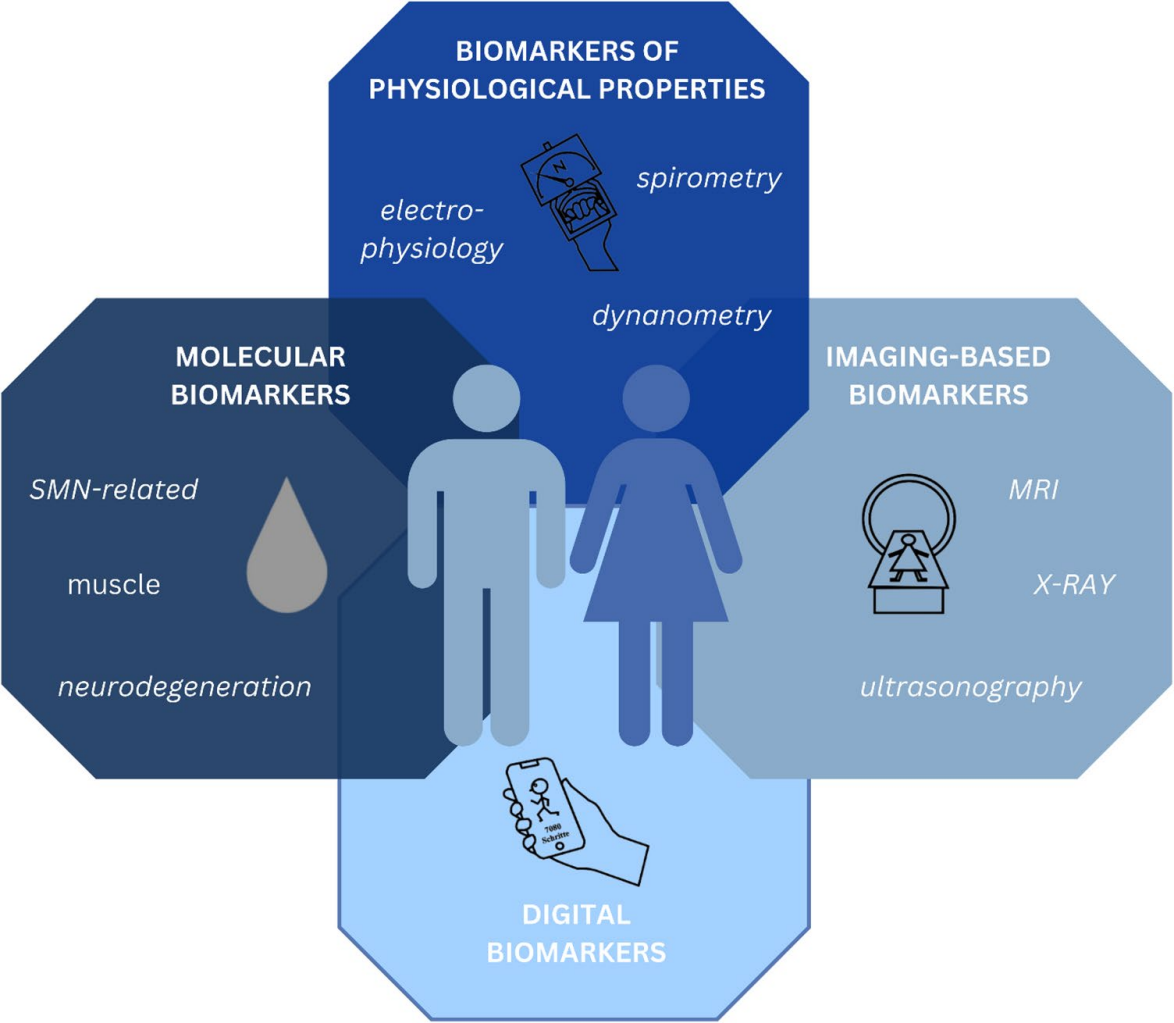
Possible biomarker sources

A lot of putative biomarker sources for SMA have been discussed, including molecular analytes, physiologic, imaging-based and clinical as well as digital biomarkers (see Fig. 2, [32, 31, 33]). Besides being valid and reliable, routine biomarkers should be easy and quick to obtain, economic and allow repeated evaluations [34]. Given their relatively easy and fast accessibility and widely available analytic techniques, the main focus will be shifting to blood-derived biomarkers. The project “Biomarkers for SMA” has been established to identify and validate such markers with a focus on correlation to motor function and other functional outcome measures. The program has identified more than 200 candidate blood-derived molecular biomarkers [35] and has proposed a plasma protein panel for SMA including 27 parameters [36]. However, the panel has not entered clinical routine yet.

To date, the in-clinic assessment of motor function as a diagnostic biomarker has been implemented in the management of SMA. However, although major efforts have been made, no physiological, imaging-based, digital or molecular biomarkers have been validated in the clinical routine in SMA patients so far. In this narrative review, we will briefly discuss previously proposed candidate biomarkers for SMA and will focus the discourse on the adoption of skeletal muscle biomarkers, especially given the muscle as a new therapeutic target.

## Biomarkers

### Molecular biomarkers

#### SMN protein (and other SMN-related biomarkers)

##### SMN protein

The amount of functional SMN-P determines the SMA phenotype with a milder phenotype in the presence of higher SMN-P levels in the ventral horn [37]. It consequently is the canonical view in the scientific community that SMN-P levels would be the most accurate biomarker for both prognostic and pharmacodynamic monitoring, especially under the systemically SMN-increasing therapy risdiplam. However, there is dispute about the best way of routinely assessing and interpreting SMN-P (compare [38]). Different groups showed that SMN-P expression varies between different tissue types [39–41]. The most easily accessible tissue for biomarkers in general is blood and a significant amount of biomarker research in SMA has been involved with measuring SMN-P and SMN-related biomarkers in the peripheral blood. SMN-P has evolved as the most promising of these biomarkers.

Some studies found that SMN-P levels in the peripheral blood relate to *SMN* copy numbers, the severity of denervation, and overall disease activity in mostly functionally more affected SMA patients [42–45] whereas different groups found neither a correlation between SMN-P blood levels and *SMN* copy numbers nor between SMN-P levels and muscle function and integrity [46, 43, 47]—or both findings at a time [43]. It is further unclear if and how blood SMN-P levels correlate with SMN-P levels in the spinal cord or the skeletal muscle [48]. SMN-P blood levels are generally lower than in the central nervous system (CNS [49]). Further, blood SMN-P levels did not increase under strictly centrally elevated SMN mRNA levels in motor neurons [50]. Given this discrepancy between central and peripheral protein levels, conclusions on central nervous processes are, therefore, limited and must be drawn carefully.

When combining the evidence from the above-mentioned studies, it becomes clear that SMN-P has the potential to serve as a diagnostic and prognostic biomarker in SMA, but that its ability to reflect dynamic processes such as disease progression or therapeutic response is still limited. It is particularly complicated as several studies suggest changing SMN-P dependency throughout the development [51] with high SMN-P levels pre- and 3 months post-natal that decrease with age [52, 53]. The cellular effects of decreasing SMN-P levels with age are not yet understood and might limit the ability to use SMN-P for disease monitoring.

With greater technical abilities and an increased understanding of pathophysiological processes in SMA, we do, however, expect enlightening research on SMN-P as a promising candidate biomarker. Its importance becomes clearer when looking at the current therapeutic options which aim at increasing SMN-P levels in the ventral horn. If a reflection of central nervous SMN-P levels can be achieved peripherally, clinical monitoring of therapy response could be supported by objective and easily accessible laboratory parameters. As a promising first step, risdiplam has been found to increase SMN-P blood levels in animal models and humans with SMA [15], indicating that this peripheral increase might reflect levels in other peripheral organs such as muscle, or the CNS [54] (see Table 1).

**Table 1:** Biomarker potential of SMN protein

Biomarker	Potential
Diagnostic	(+), conflicting data (compare [42–45])
Prognostic	(+) (compare [42–45])
Predictive	(+) (compare [42–45])
Response	+ [15]
Monitoring	(+) [15], more data needed

Potential: + high, (+) low or possible, (−) debatable, − not relevant

##### SMN mRNA transcripts (SMN2-full length and SMN transcript lacking exon 7)

Briefly, compared to controls, both parameters were decreased in the peripheral blood of patients with SMA type 1 but not in types 2 and 3 [47, 45]. Although there are conflicting data, there is no clear evidence for a correlation between *SMN* mRNA transcripts and SMA phenotypes [55] or type-specific motor function [56] (see Table 2).

**Table 2:** Biomarker potential of SMN mRNA transcripts

Biomarker	Potential
Diagnostic	(–) [47, 45]
Prognostic	(–) [55]
Predictive	Not yet investigated
Response	Not yet investigated
Monitoring	Not yet investigated

Potential: + high, (+) low or possible, (−) debatable, − not relevant

##### Limitations of SMN-derived molecular biomarkers

Although deficiency of SMN proteins and transcripts is the pathophysiological hallmark of SMA, its assessment in body fluids seems to be less useful because of only weak correlations to clinical characteristics.

#### Markers of neurodegeneration

##### Neurofilament

Neurofilaments (NFs) are tissue-specific classes of intermediate filaments that provide structural support and integrity for neurons [57]. They are released with neuronal damage and have been found elevated in numerous neurodegenerative diseases, such as amyotrophic lateral sclerosis [58, 59]. Interestingly, brain volume and change of brain volume have a relevant influence on NF levels [60]. NFs are composed of four subunits with a half-life of up to 8 months and can be assessed in both blood and cerebrospinal fluid (CSF), which makes them easily accessible markers for previous neuronal damage [31]. Especially the phosphorylated heavy chain of NFs (pNFH) and the light chain (NFL) have been studied.

In terms of SMA, elevated CSF pNFH levels have predominantly been found elevated in patients with SMA type 1 [61, 62] and specifically in those younger than 4 years [63]. In these studies, higher baseline pNFH levels correlated with early onset and lower motor function in all age groups and declined to reference levels over time. These findings were confirmed in the CHERISH study, in which pNFH levels decreased under nusinersen regardless of timepoint of treatment initiation [64]. This decline was more rapid in patients treated with nusinersen compared to those without disease-modifying therapy [65, 66], pointing to a pharmacodynamic value of pNFH. In pre-symptomatic infants with SMA, pNFH correlates with future motor milestone achievement and was the strongest predictor of motor function achievement [12].

Whereas NFs are promising biomarkers in infants with SMA, there is somewhat contradictory data in SMA types 2–4 in adolescence or adulthood. ELISA-based analyses showed no difference of serum pNFH [67] and NFL levels between healthy controls and SMA patients neither at baseline nor after treatment with nusinersen after short observation periods [68]. In CSF, NFs were similar to controls but decreasing CSF levels of pNFH and NFL during nusinersen treatment have been shown using commercially available ELISA kits [69] or the high-precision method ELLA [62], particularly in more severely affected SMA types. When corrected to muscle mass, NFL in CSF distinguished strongly between adult SMA types 2 and 3, showing higher ratios in the more severe type [62]. Therefore, normalization of NFs might be useful in progressed disease stages with already big loss of motor neurons to improve the value of these biomarkers. In another study using the highly specific single molecular array (SIMOA), no dynamic of pNFH and NFL level in CSF and serum during nusinersen treatment was reported [70]. Two studies report a negative correlation with motor function [70, 68] while one showed no correlation between NFs and motor function change [69] and one showed a positive correlation between pNFH at baseline with motor function gain under nusinersen [71].

Overall NFs seem to be reliable biomarkers for infants with SMA in terms of prognostic considerations and treatment response to nusinersen but remain unclear in adult-onset SMA or progressed disease stages in all SMA types. However, one should consider that nusinersen was first been approved in 2016 and adolescent and adult patients might have suffered from CNS motor pool exhaustion or muscular skeletal complications (e.g., contractures, scoliosis) that might have limited accurate NF assessment or reliable motor function assessment [31]. Nitz et al. hypothesized that non-neurodegenerative processes such as glia-mediated neuroinflammation or secondary myopathic changes might outweigh motor neuron loss in later stages. The age of treatment initiation might, therefore, be a more relevant determinant of NF level changes or motor function [63] and we are expecting further research on the suitability of NF levels as biomarker for later-onset SMA (see Table 3).

**Table 3:** Biomarker potential of neurofilaments

Biomarker	Potential
Diagnostic	(+) in children [61]; – in adults [70]
Prognostic	+ in children [12]; – in adults [70]
Predictive	not yet investigated
Response	+ in children [65, 69, 62, 66]; (+) in adults [69, 62, 68]
Monitoring	+ in children [65, 66]; – in adults [70], more data needed

Potential: + high, (+) low or possible, (−) debatable, − not relevant

##### Further molecular markers of neurodegeneration and neuroregeneration

Besides NFs, there are more CSF markers that are commonly associated with neurodegenerative disease, especially tau proteins and ß-amyloid peptides (Aß42 and Aß40). Both have been briefly studied in patients with SMA.

Tau protein is a microtubule-associated protein in the neuronal axons that promotes microtubule assembly and stability [72]. The presence of its phosphorylated form (pTau) is associated with aggregation and microtubule dysfunction [73]. Winter et al. found increased baseline CSF pTau values in an infant with SMA type I that decreased during nusinersen therapy [74]. These data are further supported by Johannsen et al., who replicated increased baseline pTau levels in all pediatric SMA subtypes that decreased under nusinersen [75] and by Walter et al. who reported decreased pTau levels in adults with type 3 under nusinersen [76].ß-Amyloid peptides, particularly Aß42, are assumed to play a major role in neurodegenerative diseases like Alzheimer’s disease, possibly through dysregulation of synaptic activity [77] or axonal degeneration [78]. Aß42 was not elevated in a small cohort of adult SMA patients type 2 and 3 but increased under nusinersen treatment [79] whereas other studies show stable levels of Aß42 and Aß40 under nusinersen [76].

Cathepsin D is a lysosomal aspartyl protease that is expressed in the CNS as well as skeletal and cardiac muscle and is involved in protein degradation [80]. It is believed to play a role in neurodegenerative disorders like ALS [81], Parkinson’s diseases [82] or Alzheimer’s disease [83, 84]. In patients with SMA, CSF cathepsin D levels decreased under nusinersen treatment, particularly in therapy responders, possibly due to a decreased need for protein degradation [85]. The decrease of cathepsin D was significantly correlated to a decrease in plasma NFL levels in patients aged > 12 months at the start of nusinersen treatment. As plasma NFL is a predictor of motor function achievement in children with SMA type 1 (see section on NFL), combined analyses of plasma NFL and cathepsin D might hold a prognostic potential. Cathepsin D was identified using untargeted CSF proteomic analyses, meaning the separation, identification, and quantification of the entire protein complement of the CSF. Using mass spectrometry-based unsupervised proteomic profiling in nusinersen-treated adult SMA patients, Kessler et al. identified two CSF protein clusters that differed in patient age and expression of proteins related to neurodegeneration and neuroregeneration [86]. Similarly to proteomic profiling, metabolic profiling describes the analyses of low-molecular-weight chemicals (< 1 kDa). Metabolic profiling of SMA urine samples provided specific identifiers of SMA compared to controls and was able to distinguish patients with different disease severities. First analyses even suggested the possibility to detect pre-symptomatic patients due to specific fingerprints [87]. Future untargeted analyses using highly sensitive proteomic or metabolomic profiling technologies might provide a deeper insight into SMA pathologies.

Taken together with the evidence on NFs, it is likely that neurodegenerative markers in general might reflect treatment response in SMA patients, but more data derived from bigger cohorts are needed to prove this hypothesis for tau protein, ß-amyloid peptides, and cathepsin D, and possibly other molecules not yet identified until now (see Table 4).

**Table 4:** Biomarker potential of other markers of neurodegeneration and neuroregeneration

Biomarker	Potential^a^
Diagnostic	Not yet investigated
Prognostic	(+), more data needed
Predictive	(+) [85], more data needed
Response	(+) [75, 85, 74], more data needed
Monitoring	(+) [75, 85, 74], more data needed

Potential: + high, (+) low or possible, (−) debatable, − not relevant

^a^This table gives a brief overview over tau proteins, ß-amyloid peptides and cathepsin D—for details see text section above

##### Limitations of neurodegenerative biomarkers

While markers of neurodegeneration seem promising at early stages of the disease and rapidly progressing subtypes, motor pool exhaustion might negatively affect their meaningfulness in advanced stages of the disease. There are no conclusive data on other markers than NFs yet and more investigations are warranted.

##### Glial biomarkers

An estimated 50% of cells in the human brain is constituted by glia cells, playing a crucial role in many neurodegenerative diseases.

Although the field is relatively unexplored in SMA, there is growing evidence for glia involvement in the course of the disease. Main mechanisms include increased neuroinflammation, synaptic dysregulation, and aberrant immune system activity [88]. Specifically, astrocytes have been shown to influence SMA phenotypes with therapeutically increased SMN levels in astrocytes leading to improved survival and astrogliosis being more prominent in end stage phenotypes [89]. Glial fibrillary acidic protein (GFAP) is an indicator of astroglial activity. In SMA, absolute GFAP levels did not differ from healthy controls in patients with SMA type 2 and 3, possibly due to advanced loss of motor neurons [62, 66]. When corrected to muscle mass, GFAP distinguished strongly between SMA subtypes. It might, therefore, be a candidate as complementary pharmacodynamic biomarker, reporting biological activity on non-neuronal tissue.

Chitotriosidase 1 (CHIT1) is a human endochitinase that is believed to play a role in immune system response [90] and is increased in serum and CSF in various neurodegenerative diseases such as Alzheimer’s disease or ALS [91, 92] as a marker of neuroinflammation. A recent study found elevated CSF CHIT1 levels in SMA patients that did not correlate with disease severity (SMA type, SMN copy number) or duration [93]. CHIT1 does not seem suitable to reflect disease severity, but indicates a role of neuroinflammation in the disease. Although microglial activity decreased in animal models of SMA during ASO-treatment, this observation could not be translated to treated humans [94]. In contrast, CHIT1 levels increased in response to treatment with nusinersen, independent of change in motor function [93]. This observation was supported by an independent study [95]. Taken together with observations of unknown macrophage inclusions during nusinersen therapy [96], CHIT might serve as a safety biomarker for off-target activation of innate immunity treatment but did not show pharmacodynamic potential in case of ASO treatment.

Chitinase-3-like protein 1 (CHI3L1) or YKL-40 is a chitinase that is expressed in astrocytes in neuroinflammation and is, thus, discussed as a marker of astroglial activity [97, 98]. Children with SMA type 1 showed elevated YKL-40 levels [99]. Under therapy, a decrease of YKL-40 levels was associated with improved motor function in patients with SMA type 2 and 3 [95].

S100B is a calcium-binding protein that is mainly found in glial cells and supports cell survival [100]. It is a marker of glial cell death and has mainly been studied in traumatic brain injuries [101], but its overexpression has also been related to active neuronal distress in neurodegenerative disorders like Alzheimer’s disease or ALS [102]. In SMA, S100B was slightly elevated in 1 out of 11patients in serum and CSF [67]. More data are warranted.

Although data on glial biomarkers in SMA are scarce, some of them might be valuable as diagnostic and pharmacodynamic biomarkers, particularly as they might reflect neuroinflammation. The relevance might be higher in more rapidly progressive SMA subtypes (see Table 5).

**Table 5:** Biomarker potential of glial biomarkers

Biomarker	Potential
Diagnostic	(–) [93, 62]
Prognostic	Not yet investigated
Predictive	Not yet investigated
Response	(+) [95, 62]
Monitoring	Ranging from – [93] to (+) [95]

Potential: + high, (+) low or possible, (−) debatable, − not relevant

##### Limitations of glial markers

Despite some promising insights in glial pathology in SMA, the existing data do not allow a conclusive routine exertion of glial biomarkers in clinical SMA management yet.

#### Skeletal muscle biomarkers

SMN-P is an ubiquitously expressed protein and, as a consequence, disturbances in multiple peripheral organs have been described in the past years. Schreml et al. found abnormalities in intestine, heart, lung, and skeletal muscle vasculature in SMA mice [103] and in humans, there are numerous reports on cardiac and autonomous defects (for example, see [104–106]) as well as liver and metabolic disorders (for an overview, see [107]). Accordingly, SMA has been considered to be a multi-systemic disease [108].

Regarding the skeletal muscle, there is growing evidence that SMN depletion leads to aberrant muscle and NMJ development in SMA mice [109–113] and aberrant muscle structure and function in human SMA patients have been confirmed (see chapter on electrophysiological biomarkers and [103, 108, 114–116]).

Skeletal muscle regulates synaptic activity and axonal function of motor neurons and provides trophic support [117]. Although SMA is primarily a motor neuron disorder, secondary skeletal muscle atrophy and myopathy, developmental alterations of the postsynaptic motor endplate and neurodegeneration of other extra-motor tissues have been well described.

Given this muscular involvement in the pathogenesis and clinical features in SMA, skeletal muscle laboratory parameters have recently become of interest.

##### Creatinine and creatine kinase

Creatine and the creatine kinase (CK) play an important regulatory role in the energy metabolism in cells with high energy demand by ensuring restoration of adenosine triphosphate levels [118]. While there are small concentrations of creatine and CK in cardiac and smooth muscle, brain, and other tissues, more than 90% of creatine are stored in the skeletal muscle [119]. The creatine metabolism, therefore, is essential for muscle function and integrity. Creatinine (Crn) is a metabolite from skeletal muscle creatine metabolism and the measurable parameter of this process. Research in other neurodegenerative diseases has shown decreased Crn levels and increased CK levels in spinal and bulbar muscular atrophy (SBMA, [120, 121]) and ALS [122, 123]. Crn is generally considered a marker of muscle mass and CK a marker of muscle damage.

Similarly to SBMA, two studies report increased CK levels and decreased Crn levels in SMA patients compared to healthy controls, with Crn levels able to distinguish clearly between SMA subtypes in children and adults [124, 125]. They were further related to motor function and denervation as measured with CMAP and MUNE. When corrected for weight, height, and age, baseline CK und Crn levels in treatment-naïve patients differed between responders and non-responders to nusinersen and under treatment, CK decreased and Crn increased, reflecting reduced muscle wasting and improved muscle energy metabolism [125]. Crn is detectable in urine samples, and pilot studies report significant differences between SMA patients and healthy controls [126]. Whether or not Crn might serve as a prognostic, predictive or monitoring biomarker in urine is still to be investigated. The study has a short observation period, in which no changes from baseline were observed [126]. A non-invasive way of Crn assessment is, however, intriguing as blood sample collection can be difficult in some SMA patients. Although data are limited, Crn and CK are promising candidate biomarkers for disease progression with and without therapy in SMA patients. As oral Crn supplementation can increase muscular Crn content and thereby possibly improve muscle function [35], therapeutic effects of Crn have been studied in numerous neurodegenerative disorders and myopathies [127, 128]. Wong et al. reported no significant improvement of motor function, muscle strength or quality of life in children with SMA after 6 months of oral Crn [129]. As this is to the best of our knowledge, the only study on oral Crn in SMA patients, future research including larger cohorts and different dosages should be performed (see Table 6).

**Table 6:** Biomarker potential of creatinine and creatine kinase

Biomarker	Potential
Diagnostic	+ [124, 125]
Prognostic	(+) [124, 125]
Predictive	(+) [125]
Response	+ [124, 125]
Monitoring	(+) [124, 125]

Potential: + high, (+) low or possible, (−) debatable, − not relevant

##### Other potential skeletal muscle biomarkers

Given the fact that Crn and CK are promising biomarkers for monitoring disease progression and diagnostic purposes in patients with SMA, and that the skeletal muscle is more and more evolving as a diagnostic and therapeutic target, we consider investigating other potential skeletal muscle biomarkers as important and meaningful in the quest for reliable SMA biomarkers.

#### Myoglobin

Myoglobin (Myo) is a cytoplasmatic hemoprotein that is solely expressed in cardiac myocytes and oxidative skeletal muscle fibers. It is an important oxygen storage that can release oxygen in case of hypoxia or anoxia, buffer muscular oxygen concentrations. And facilitate oxygen diffusion in activated muscle with the purpose of providing equivocal oxygen levels in any degree of muscle activity [130]. Similar to CK, Myo is released in case of muscle damage or impaired integrity of muscle cell membrane and might serve as a marker for muscle wasting and aberrant muscle metabolism in motor neuron diseases. In SBMA, Myo was significantly elevated compared to ALS patients and related with disease progression [131]. To the best of our knowledge, there are no studies on Myo in SMA yet. However, based on the above-mentioned findings, we propose Myo as a potential SMA biomarker (see Table 7).

**Table 7:** Biomarker potential of myoglobin

Biomarker	Potential
Diagnostic	Not yet investigated
Prognostic	Not yet investigated
Predictive	Not yet investigated
Response	Not yet investigated
Monitoring	Not yet investigated

Potential: + high, (+) low or possible, (−) debatable, − not relevant

#### Troponin T

Troponins are involved in the calcium regulation in striated muscle and essential for muscle structure and function [132]. There are several troponin isoforms of which Troponin I (TNI) and Troponin T (TNT) are generally considered highly specific for cardiac muscle [133]. However, there is an increasing number of non-cardiac diseases that are associated with TNT elevation, particularly neurodegenerative disorders [134]. A secondary cardiac involvement has previously been proposed. In a recent study in ALS, Castro-Gomez et al. found elevated TNT levels in ALS patients without TNI elevation or clinical signs of cardiac involvement, and thus proposed an extracardiac origin of TNT with TNT serving as a biomarker for lower motor neuron affection [135]. Although SMA and ALS have fundamentally different pathogenesis, they do partially share identical phenotype and pathomechanisms [136]. Changes of troponin isoform expression in muscles of SMA patients are described [137]. An increased understanding of the role of TNT in SMA is especially important as novel troponin activating therapies are in the pipeline [27] (see Table 8).

**Table 8:** Biomarker potential of Troponin T

Biomarker	Potential
Diagnostic	Not yet investigated
Prognostic	Not yet investigated
Predictive	Not yet investigated
Response	Not yet investigated
Monitoring	Not yet investigated

Potential: + high, (+) low or possible, (−) debatable, − not relevant

##### Limitation of skeletal muscle biomarkers

Skeletal muscle biomarkers comprise markers of muscle destruction and integrity, that can add valuable information in the therapeutic management of SMA when combined. They are, however, dependent on individual factors such as body and muscle mass, renal function, and (cardiac) comorbidities and must, therefore, be interpreted carefully.

##### Biomarkers of physiological properties

*Electrophysiology* Electrophysiological measurements can monitor the functional status of the motor unit pool and are, therefore, extremely important in the diagnostic process and monitoring of disease progression in motor neuron disorders. compound muscle action potential (CMAP), motor unit number estimation (MUNE), and motor unit number index (MUNIX) have evolved as the most reliable measures.

#### CMAP

Reduction of CMAP amplitudes indicate a reduction of motor units supplying a particular muscle or group of muscles [31]. Arnold et al. found significantly reduced CMAP in 2-week-old SMA mice, that could be restored under SMN-increasing therapy [138]. In humans, CMAP of the ulnar nerve was reduced in SMA patients and correlated with SMA type, age, and *SMN2* copy number [139–141]. It improved in children treated with onasemnogene abeparvovec [142] and nusinersen [143] and stayed stable during nusinersen treatment [144]. Patients with a faster post-treatment ulnar CMAP-increase tended to have better functional outcome [141]. These data suggest that CMAP is a reliable marker of disease progression under therapy in SMA children and is especially suitable for children as it does not require cooperation of the patient. It might further be an early indicator of the degree of therapy response in children with SMA and underlines the importance for an early therapy start in patients with SMA, regardless of the clinical subtype. However, further research is needed as some data suggest that CMAP might not adequately reflect motor neuron loss in only lightly affected muscle groups [145] (see Table 9).

**Table 9:** Biomarker potential of CMAP

Biomarker	Potential
Diagnostic	+ [140, 141]
Prognostic	(+) [140, 141]
Predictive	(+) [141]
Response	+ [142, 143]
Monitoring	+ [144]

Potential: + high, (+) low or possible, (−) debatable, − not relevant

#### MUNE

While CMAP measures the electrical output of a muscle, MUNE estimates the number of innervating motor neurons [31]. Similarly to CMAP, it is reduced in patients with SMA type 1 and 2, correlated with SMA type, age, and *SMN2* copy number [140, 141] and improved in children treated with onasemnogene abeparvovec [142] and nusinersen [143]. In adult SMA type 3 patients, MUNE was decreased compared to controls but did not change under nusinersen [146]. MUNE holds great potential as a biomarker for diagnostic measures and also promising value for prognostic and pharmacodynamic purpose. However, further research is needed as follow-up data on children with SMA type 3 show decreasing long-term MUNE measures, despite therapy [144] and data in adult SMA patients are scarce (see Table 10).

**Table 10:** Biomarker potential of MUNE

Biomarker	Potential
Diagnostic	+ [140, 141]
Prognostic	(+) [140, 141]
Predictive	Not yet investigated
Response	(+) [11, 23]
Monitoring	+ [142, 141]

Potential: + high, (+) low or possible, (−) debatable, − not relevant

#### MUNIX

Assessing the motor unit number index (MUNIX) is a non-invasive technique that estimates the number of functional motor units innervating a particular muscle or group of muscles [147]. It has been well established in ALS [147, 148] as a reliable and valid marker for motor unit loss. Recent studies in patients with SMA showed relevant correlations of MUNIX and motor function and disease progression in both adults and children [139, 145, 149, 150]. MUNIX further detects disease-specific patterns of motor unit loss, allowing a precise distinction of SMA versus ALS patients [139]. Summed up, MUNIX seems to be a suitable biomarker for monitoring disease progression in the natural course of the disease and possibly under disease-modifying therapies (see Table 11).

**Table 11:** Biomarker potential of MUNIX

Biomarker	Potential
Diagnostic	(+) [139]
Prognostic	+ [149, 145, 150]
Predictive	Not yet investigated
Response	Not yet investigated
Monitoring	Not yet investigated

Potential: + high, (+) low or possible, (−) debatable, − not relevant

#### Other electrophysiological markers

#### MUSIX

Motor unit size index (MUSIX) can be calculated with MUNIX and CMAP amplitudes and provides information on the size of motor units of a particular muscle or muscle groups [151]. They show increased values in SMA patients, suggesting re-innervation [145]. Investigations of the relevance of MUSIX as a biomarker in SMA are scarce, but MUSIX might add valuable information for the monitoring of disease progression with and without therapy. However, more research needs to be done (see Table 12).

**Table 12:** Biomarker potential of MUSIX

Biomarker	Potential
Diagnostic	Not yet investigated
Prognostic	Not yet investigated
Predictive	Not yet investigated
Response	Not yet investigated
Monitoring	Not yet investigated

Potential: + high, (+) low or possible, (−) debatable, − not relevant

#### EIM

Electrical impedance myography (EIM) is a relatively new, easy, and painless technique which provides information on the impedance characteristics of the underlying muscle. It showed a good correlation with muscle strengths in children with SMA type 2 and 3 [152] and might be able to discriminate between healthy subjects and those with SMA [153]. Animal models further show correlation to SMN protein levels and CMAP data [154] (see Table 13).

**Table 13:** Biomarker potential of EIM

Biomarker	Potential
Diagnostic	(+) [153]
Prognostic	+ [152]
Predictive	Not yet investigated
Response	Not yet investigated
Monitoring	Not yet investigated

Potential: + high, (+) low or possible, (−) debatable, − not relevant

##### Repetitive nerve stimulation

Animal models have suggested early disturbances of the neuromuscular junction (NMJ) in SMA pathology [155–157]. It is unclear whether or not SMN-increasing therapy has beneficial effects on the NMJ integrity or function. Arnold et al. used repetitive nerve stimulation to detect NMJ transmission defects in adult SMA patients [158]. The defects were not corrected after 14 months of nusinersen treatment and correlated negatively with motor function. Although data on repetitive nerve stimulation and NMJ deficits in SMA in general are rare, studying the NMJ in SMA appears to be a promising field for establishing new therapeutic approaches and possibly also biomarkers, especially in the context of new muscle-targeted therapeutic approaches. In a phase 2 study, Bonanno et al. found improved motor function in ambulatory SMA type 3 patients treated with amifampridine, which improves NMJ transmission [29] (see Table 14).

**Table 14:** Biomarker potential of repetitive nerve stimulation

Biomarker	Potential
Diagnostic	Not yet investigated
Prognostic	Not yet investigated
Predictive	(+) [158]
Response	Not yet investigated
Monitoring	(+) [158, 29]

Potential: + high, (+) low or possible, (−) debatable, − not relevant

#### Limitations to electrophysiological biomarkers

The above-mentioned electrophysiological methods partly reflect the integrity and function of the motor unit in SMA patients, both adults and children suggesting a high biomarker potential. However, there are some considerable limitations: Some of the techniques are painful (e.g., CMAP, MUNE, MUNIX) and/or require the cooperation of the patient (e.g., MUNIX, spirometry) and might, thus, not be suitable for infants and children. While spirometry shows promising diagnostic, prognostic, and monitoring properties and is well established in the clinical routine, the electrophysiological techniques might be more difficult to be implemented routinely as they are resource and time consuming, exhibit inter-rater variability, and need intensive training.

#### Functional biomarkers

##### Dynamometry

Muscle strength of SMA patients is usually assessed using the Medical Research Council (MRC) scale that ranges from 0 (no muscle contraction at all) to 5 (normal strength) [159]. However, the scale requires extensive training, lacks sensitivity in very weak muscles and displays high intra- and inter-rater variability [160–162]. Merlini et al. showed reliable measurements of muscle strength in SMA with a hand-held dynamometer [163] and linked muscle strength to motor function [164]. Under nusinersen, handgrip strength significantly improved in patients with SMA type 3 and 4 and correlated with motor function change in the RULM [165]. Seferian et al. further investigated grip and pinch strength in patients with SMA type 2 and 3 and found significantly lower muscle strength in type 2 patients [166]. Interestingly, both grip and pinch strength increased in young non-ambulatory patients (< 14 years of age) but declined in older patients [166]. A possible explanation is an inability of growth to compensate for strength loss in older patients. Dynamometry is a non-invasive tool that seems to be suitable for the implementation in clinical routine to assess and monitor motor function in SMA patients (see Table 15).

**Table 15:** Biomarker potential of dynamometry

Biomarker	Potential
Diagnostic	(+) [165, 166]
Prognostic	Not yet investigated
Predictive	Not yet investigated
Response	Not yet investigated
Monitoring	(+) [165, 166]

Potential: + high, (+) low or possible, (−) debatable, − not relevant

##### Spirometry

Respiratory dysfunction is a crucial cause of death in neuromuscular diseases including SMA, particularly in more severely affected patients [167]. Pulmonary function usually declines throughout the natural cause of the disease, often leading to the need for ventilatory support or tracheotomy [168]. Both the forced expiratory volume in 1 s (FEV1) and forced vital capacity (FVC) differ between SMA subtypes and decline over time [169]. Under nusinersen, multiple studies report a stable or even improved pulmonary function and an higher likelihood of event-free survival (e.g., free from tracheotomy or ventilation assistance) in SMA types 1 and 2 [170–172] whereas a few do not [173, 174] (see Table 16).

**Table 16:** Biomarker potential of spirometry

Biomarker	Potential
Diagnostic	(+) [169]
Prognostic	(+) [169]
Predictive	Not yet investigated
Response	Not yet investigated
Monitoring	(+) [170, 171, 174]

Potential: + high, (+) low or possible, (−) debatable, − not relevant

### Imaging-based biomarkers

#### Quantitative MR imaging

##### Muscle

Quantitative magnetic resonance imaging (qMRI) is a device-based tool for muscle physiology assessment.qMRI of muscles has been performed in several neuromuscular diseases, for example Charcot–Marie–Tooth disease, inclusion body myositis [175] or Duchenne muscular dystrophy [176]. Especially in Charcot–Marie–Tooth disease, it has proven to correlate with motor function scales and disease severity at baseline and longitudinally.

In patients with SMA, Bonati et al. demonstrated a reduced muscle mass and density of the thigh muscle with an increased infiltration by fatty tissue. Similarly, Wadman et al. reported fatty degeneration of mouth opening muscles and a sign of bulbar dysfunction in SMA [177] and fatty degeneration of muscles of upper and lower extremity muscles are well described [178–181]. Further cross-sectional evaluations of qMRI indices were able to clearly distinguish between SMA patients and healthy participants but it remains unclear if a stratification among SMA types is feasible [46]. Muscle volume and other qMRI markers in SMA patients correlated strongly with impairment of motor function in several studies; however, these markers did not seem to have a prognostic potential [46, 180, 178–183]. qMRI measures were associated with decrease of motor function in a recent study. However, no changes were observed during 6 months [181, 185] or 14 months [107] of nusinersen treatment whereas motor function measured with motor scores increased [107], arguing for a limited monitoring biomarker potential. In treatment-naïve patients, Otto et al. reported a slow disease progression in qMRI despite sustained motor function and muscle strength [179]. These somewhat contradictory findings leave room for further investigation of the utility of qMRI as a biomarker in SMA, especially as a monitoring biomarker. As of today, qMRI is a non-invasive and well-tolerable method to add valuable information on morphological characteristics of the effector tissue muscle, unaffected by influencing factors such as patient’s fatigue or daily fluctuations [46]. It is, however, relatively expensive and can currently not be used as a stand-alone biomarker. Nonetheless, qMRI will be of interest for further investigations, especially in the context of new muscle-targeting drugs (see Table 17).

**Table 17:** Biomarker potential of qMRI

Biomarker	Potential
Diagnostic	(+) [46]
Prognostic	(–) [46, 184, 183]
Predictive	Not yet investigated
Response	Not yet investigated
Monitoring	– [185]

Potential: + high, (+) low or possible, (−) debatable, − not relevant

##### Nerve

Quantitative MR neurography (qMRN) is a 3 Tesla MRI-based technique that enables high-resolution visualization of both the peripheral nervous system and the musculature in one step. One important strength of qMRN is its ability to assess even the most proximal nerve segments, i.e., spinal nerves and plexus, that are hardly accessible through traditional electrophysiologic methods but are of special interest in SMA [186]. qMRN has proven to detect PNS lesions with high sensitivity in a wide variety of PNS-affecting diseases [187, 188], Similarly, qMRN detected and quantified PNS lesions in therapy-naïve patients with SMA types 2, 3a, and 3b in vivo [189]. This work also defined qMRN parameters (T2 relaxation time, proton spin density, cross-sectional area) that might serve as imaging biomarkers in SMA to indicate early microstructural nerve tissue changes. One particular parameter, the magnetization transfer ratio, differed between SMA type 3 patients and healthy controls and correlated with clinical scores [190]. Further research is needed to determine the value of these qMRN markers in evaluating response to SMA treatments and diagnostic procedures [189, 190].

Central nervous quantitative MR imaging has exposed cervical spinal cord atrophy in treatment-naïve adult SMA patients compared to healthy controls [191] and these changes might be based on gray matter atrophy [192, 193]. A longitudinal pilot study with three adult SMA patients found stable gray matter volumes in two out of three patients under nusinersen and increasing volumes in the third patient [193]. Thus, spinal cord and particular gray matter volume are candidate diagnostic, response and monitoring biomarkers. However, a lot more research in larger cohorts is warranted (see Table 18).

**Table 18:** Biomarker potential of qMRN

Biomarker	Potential
Diagnostic	(+) [189]
Prognostic	(+) [189], more data needed
Predictive	Not yet investigated
Response	(+) [193]
Monitoring	(+) [193]

Potential: + high, (+) low or possible, (−) debatable, − not relevant

#### Ultrasonography

##### Muscle

Muscle ultrasonography is a non-invasive and relatively inexpensive tool in the diagnostics of neuromuscular disorders. It has proven its worth in different diseases involving muscle atrophy, for example Duchenne muscular dystrophy [194–196]. Different diseases show different ultrasonography changes with SMA showing an inhomogeneous increase of echo intensity with severe atrophy of the muscle [195]. A small pilot study reported feasibility of discrimination between healthy controls and SMA patients [197], but this study was limited by a small sample size and many possible confounding factors (e.g., technical factors involving the ultrasonography machine and high inter- and intra-rater variability) still limit the usability of this method (see Table 19).

**Table 19:** Biomarker potential of muscle ultrasonography

Biomarker	Potential
Diagnostic	(+) [198, 197]
Prognostic	Not yet investigated
Predictive	Not yet investigated
Response	Not yet investigated
Monitoring	Not yet investigated

Potential: + high, (+) low or possible, (−) debatable, − not relevant

##### Nerve

Similar to muscle ultrasonography, nerve ultrasonography is a non-invasive and relatively inexpensive diagnostic tool. To this day, there is one small study on ultra-high-frequency nerve ultrasonography in patients with SMA [199]. The authors investigated nerve area and fascicle density and number in the median nerve of one patient with each SMA type 1, 2, and 3 in comparison to healthy controls. While there were no differences between controls and SMA type 2 and 3, the patient with SMA type 1 showed a reduced fascicle number, possibly related to the rapid clinical progress in this subtype. The study is, however, too small for conclusive information (see Table 20).

**Table 20:** Biomarker potential of nerve ultrasonography

Biomarker	Potential
Diagnostic	(+) [199]
Prognostic	Not yet investigated
Predictive	Not yet investigated
Response	Not yet investigated
Monitoring	Not yet investigated

Potential: + high, (+) low or possible, (−) debatable, − not relevant

##### Diaphragm

Diaphragmatic ultrasound has found diaphragmatic dysfunction in adult SMA patients that correlates with the FEV1 and FVC [200–202]. Pathological findings mainly include reduced contractility but not atrophy with differential data for adults and children (compare [202]). It is an easy and non-invasive tool for the assessment of pulmonary function, that does not require the patient’s compliance and is, thus, also suitable for children. To this day, the clinical benefit over spirometric parameters remains unclear (see Table 21.

**Table 21:** Biomarker potential of diaphragmatic ultrasound

Biomarker	Potential
Diagnostic	(+) [202]
Prognostic	(+) [200]
Predictive	Not yet investigated
Response	Not yet investigated
Monitoring	Not yet investigated

Potential: + high, (+) low or possible, (−) debatable, − not relevant

#### Dual-energy X-ray absorptiometry

Measuring body composition (BC) with dual-energy X-ray absorptiometry has been used for monitoring disease severity and progression in patients with neuromuscular disorders [203]. Baranello et al. investigated relevant BC parameters like body weight, supine lengths, body mass index, fat and lean mass as well as composite measures consistent of bone mineral content and lean mass (“FFM”) in correlation to motor function [204]. The study revealed that a good body composition is associated with good motor function, which underlines the importance of monitoring the nutritional status of SMA patients. Furthermore, lean body mass might be a co-factor when interpreting molecular biomarkers of muscle integrity [124]. The authors themselves, however, recognize that further research should be undertaken considering relatively low correlations (see Table 22).

**Table 22:** Biomarker potential of dual-energy X-ray absorptiometry

Biomarker	Potential
Diagnostic	Not yet investigated
Prognostic	(+), more data needed
Predictive	Not yet investigated
Response	Not yet investigated
Monitoring	Not yet investigated

Potential: + high, (+) low or possible, (−) debatable, − not relevant

#### Limitations of imaging-based biomarkers

Imaging-based biomarkers reflect the current muscle and nerve structure in SMA patients. However, they have not been proven to be of use in monitoring disease courses in SMA with or without treatments (monitoring biomarker). Promising data come from central nervous quantitative MRI imaging. The techniques are costly and standardized protocols are not yet widely available. To date, their routine clinical use remains limited.

### Digital biomarkers

Electronics and digital devices have long become a substantial part of our daily private and professional life. In SMA, they facilitate participation and are an important part of the patient’s autonomy. It is only consistent to investigate the use of digital technology in the SMA management. Two studies have investigated the suitability of different movement sensors for the assessment of upper limb function in SMA patients. Chen et al. used the Microsoft Kinect sensor to record movements of patients with SMA type 3 and compared them to healthy controls [205]. Although the sensor distinguished between controls and patients at later stages of the disease, it was not (yet) able to distinguish early-stage differences. Chabanon et al. used the motor sensor ActiMyo to measure upper limb function and found that numerous parameters correlated to motor function as measured with the common scales and differed between SMA subtypes [206]. More app-based tools are currently under investigation. One example is the Konectom app that includes the assessment of upper and lower limb motor function [207].

Overall, digital outcome assessments hold the strong potential to monitor motor function in SMA patients in the future, but more research is warranted. They are particularly interesting as they might provide valuable insight on motor function in the patients’ everyday life and fluctuations during the day.

Classification of digital biomarkers is challenging. They measure physiological functions and could, therefore, be listed under biomarkers of physiological properties. We did, however, distinguish them from conventional (electro-)physiological measures due to the novelty of the used technology and appliances and the fact that they can be used remotely outside the clinic (see Table 23).

**Table 23:** Biomarker potential of digital biomarkers

Biomarker	Potential
Diagnostic	(+) [206, 205]
Prognostic	Not yet investigated
Predictive	Not yet investigated
Response	Not yet investigated
Monitoring	Not yet investigated

Potential: + high, (+) low or possible, (−) debatable, − not relevant

## Conclusion

The interest in biomarkers for SMA has grown over the past decade, alongside with a change of research focus from basic to clinical research. Driver of this paradigm shift has not at least been the development of new disease-modifying therapies and a concomitant need for parameters that help clinicians navigate through the therapeutic process.

Research on suitable biomarkers has been extensive and broad, but a lot of work still remains to be done. Many multimodal potential biomarkers for diagnostic, prognostic, and predictive purposes have been identified; however, none of them have (yet) emerged as one single overarching marker.

Circulating SMN-related biomarkers (SMN protein, particularly SMN2 copy number) have a good diagnostic value and hold the potential to monitor therapy response in the future. Markers of neurodegeneration such as NFs have proven to be good prognostic markers and monitor therapy response in SMA patients. Skeletal muscle-associated plasma proteins (Crn, CK) can predict treatment response and further accurately monitor disease progression and motor function. Similar routine parameters (TropT and Myo) show promising potential in other neurodegenerative diseases and could be of additional predictive value to the monitoring of patients with SMA; however, pending investigations need to be done. Electrophysiological parameters such as CMAP, MUNE, and MUNIX developed as trustworthy measures of motor function in patients with SMA during the course of the disease and allow prediction of treatment effect. Imaging-based techniques involving MRI und ultrasonography can distinguish between healthy subjects and SMA patients and provide insight in morphological properties of muscle and nerve during disease course; however, difficulties in the clinical routine are expected due to cost in relation to other measures, inter- and intra-rater variability, and limited ability. Digital biomarkers can be easily implemented in the patient’s daily routine and can assess motor function under real-world conditions. As telemedicine and e-health are progressively established in health care, the importance of digital biomarkers is likely to grow in the next years.

Different biomarkers are needed for different purposes. As laid out above, none of the markers can sufficiently meet all the expected criteria. A combination of different biomarkers with clinical assessments at different times in the course of the disease appears to be the most comprehensive solution at the time. Besides the obligatory genetic testing and clinical assessment, a possible biomarker scheme could include baseline acquisition of SMN2 copy number, electrophysiology (CMAP), plasma protein levels of SMN protein, NF, Crn, CK, and CSF NF protein levels with regular follow-ups on fluid and digital biomarkers and annual or biennial follow-ups on the electrophysiological markers (see Table 24).

**Table 24:** Overview of the discussed biomarkers

	Biomarker potential				
Biomarker	Diagnostic	Prognostic	Predictive	Response	Monitoring
Molecular biomarkers					
SMN protein	(+)	(+)	(+)	+	(+)
SMN mRNA transcripts	(–)	(–)	nyi	nyi	nyi
Neurofilaments	(+) in children; – in adults	+ in children; - in adults	nyi	+ in children; (+) in adults	+ in children; – in adults
Markers of neurodegeneration and neuroregeneration	nyi	(+)	(+)	(+)	(+)
Glial biomarkers	(–)	nyi	nyi	(+)	ranging from—to (+)
Creatinine and creatine kinase	+	(+)	(+)	+	(+)
Myoglobin	nyi	nyi	nyi	nyi	nyi
Troponin T	nyi	nyi	nyi	nyi	nyi
Biomarker of physiological properties					
CMAP	+	(+)	(+)	+	+
MUNE	+	(+)	nyi	(+)	+
MUNIX	(+)	+	nyi	nyi	nyi
MUSIX	nyi	nyi	nyi	nyi	nyi
EIM	(+)	+	nyi	nyi	nyi
Repetitive nerve stimulation	nyi	nyi	(+)	nyi	(+)
Dynamometry	(+)	nyi	nyi	nyi	(+)
Spirometry	(+)	(+)	nyi	nyi	(+)
Imaging-based biomarkers					
qMRI	(+)	(–)	nyi	nyi	-
qMRN	(+)	(+)	nyi	(+)	(+)
Muscle ultrasonography	(+)	nyi	nyi	nyi	nyi
Nerve ultrasonography	(+)	nyi	nyi	nyi	nyi
Diaphragmatic ultrasound	(+)	(+)	nyi	nyi	nyi
Dual-energy X-ray absorptiometry	nyi	(+)	nyi	nyi	nyi
Digital biomarkers					
Digital biomarkers	(+)	nyi	nyi	nyi	nyi

Biomarker potential: + high, (+) low or possible, (−) debatable, − not relevant; nyi = not yet investigated. For sources see individual tables

## Data Availability

There are no data to disclose as this work is a narrative review.
